# Assessment of the bacterial community of the human upper respiratory tract in patients affected by Covid-19

**DOI:** 10.1590/1678-4685-GMB-2025-0076

**Published:** 2026-06-26

**Authors:** Daniel Guasselli, Sávio Costa, Gleyciane Costa, Rennan Moreira, Guilherme Baião, Rafael Baraúna, Artur Silva, Diego Graças

**Affiliations:** 1 Universidade Federal do Pará, Laboratório de Engenharia Biológica, Belém, PA, Brazil.; 2 Universidade Federal de Minas Gerais, Departamento de Microbiologia, Instituto de Ciências Biológicas, Laboratório de Biologia Molecular e Computacional de Fungos, Belo Horizonte, MG, Brazil.; 3 Universidade Federal de Minas Gerais (UFMG), Instituto de Ciências Biológicas, Centro de Laboratórios Multiusuários, Laboratório de Genômica, Belo Horizonte, MG, Brazil.

**Keywords:** 16S, NGS, SARS-CoV2, Microbial ecology, human microbiota

## Abstract

Given the challenges posed by the 2019 coronavirus disease (COVID-19), understanding the role of the microbiota is crucial. The analysis of this microbial community in the body not only expands our understanding of health, but also provides insights into the interaction between viruses, microbiota, and the human host. In this study, we aimed to identify possible variations in bacterial diversity caused by Severe Acute Respiratory Syndrome Coronavirus 2 (SARS-CoV-2). The study involved 45 volunteers, divided into the Positive Group (PG) consisting of 14 patients and the Negative Group (NG) consisting of 31 individuals. Both groups were stratified by biological sex to ensure sample homogeneity. Samples were collected using nasopharyngeal and oropharyngeal swabs, and total DNA was extracted and stored. The V4 region of the 16S rRNA gene was sequenced, and bioinformatics tools were used to assess the composition and diversity of the respiratory microbiota. In our results, the most abundant bacterial phyla were: Firmicutes, Bacteroidetes, and Proteobacteria. There was a notable reduction in the frequency of Firmicutes in the PG, suggesting a potential compromise in the immune response to viral infection. Beta diversity analysis did not reveal significant variations between the communities of the groups. Additionally, the analysis indicated subtle changes in some taxa, such as an increase in the abundance of Neisseriaceae in patients affected by COVID-19. These findings contribute to a deeper understanding of the complex interaction between the virus, the microbiota, and the host during infection with SARS-CoV-2, highlighting the importance of the respiratory microbiota in the context of COVID-19.

## Introduction

The viral agent SARS-CoV-2, responsible for Acute Respiratory Syndrome (COVID-19), caused the recent pandemic that began in 2019 and presented global-scale challenges (Lotfi et al., 2020). Respiratory disease is mainly transmitted through droplet pathways or secondarily through fomites. Shortness of breath, fever, dry cough, headache, and pneumonia are the main manifestations of the disease, with variations related to the vulnerability of individuals, such as neurological, cardiac, and renal symptoms. In severe cases, it can cause respiratory distress and be fatal (Atzrodt et al., 2020; Canedo-Marroquín et al., 2020). 

SARS-CoV-2 and other respiratory pathogens primarily infect the nasopharyngeal epithelium, which serves as the entry point into the human body and forms part of the upper respiratory tract (URT). With tropism for the respiratory tract and diverse manifestations, similar to other viruses, SARS-CoV-2 can migrate and lodge in the Lower Respiratory Tract (LRT), thus promoting inflammation that triggers the respiratory syndrome (Clementi et al., 2021; Chiu et al., 2022).

The upper respiratory tract performs essential physiological functions, including warming, humidifying, and filtering inspired air (Watelet and Cauwenberge, 1999). Within the URT, viruses encounter both epithelial cells and commensal microbes that maintain symbiotic relationships with the host. The human microbiome is a complex microbial community that can vary depending on the tissue and environmental stimuli. The nasopharynx provides a variety of niches and habitats for specific microbial colonization, as it presents a series of local environmental conditions that support bacterial proliferation. 

These communities are shaped by both host characteristics (age, immune status, pathologies) and external factors (mode of delivery, lifestyle habits, environmental exposures) (Costello et al., 2009; Shilts et al., 2016; Kumpitsch et al., 2019). Additionally, microbial communities modulate the immune defense with URT cells, developing crucial characteristics for host defense against pathogens. Thus, the mechanism of human respiratory health involves the complex interaction between the microbiota and the host (Stearns, 2015; Man et al., 2017).

The respiratory tract microbiota can undergo ecological disturbances due to various stimuli, and anatomical or environmental effects, which consequently cause a reduction or increase in bacterial growth and diversity. Therefore, some distinct conditions and microenvironments can be transient or resident (De Steenhuijsen Piters et al., 2015; Copeland et al., 2018). Considering healthy adult human individuals, the bacterial community of the human upper respiratory tract (nasopharynx and oropharynx) is predominantly represented by the phyla Actinobacteria, Bacteroidetes, Firmicutes, and Proteobacteria, and by the genera *Corynebacterium*, *Staphylococcus*, and *Streptococcus.* In addition to the presence of various healthy commensals, including *Veillonella* and *Porphyromonas*, which can trigger the immune response and alter susceptibility to viral infection, they also serve as biomarkers for control individuals in other studies (Stearns, 2015; Kumar et al., 2022)*.*

The colonization of the bacterial community in the upper respiratory tract plays an essential role in predisposing individuals to respiratory tract infections. Understanding this process is crucial for identifying the evolutionary pressures that shape the virulence and pathogenesis of respiratory diseases. This bacterial colonization in the upper respiratory tract is a crucial element in determining susceptibility to respiratory infections, providing valuable insights into the evolutionary influences affecting the severity and progression of these conditions (Bosch et al., 2013; Siegel and Weiser, 2015).

Respiratory viral infections, such as influenza, can disrupt host microbial interactions, increasing the risk of secondary bacterial pneumonia. This imbalance is crucial in the progression and severity of the condition, contributing to a deeper understanding of the pathogenesis associated with these respiratory infections. Coinfection with bacteria causing atypical pneumonia is critical in COVID-19 patients due to the potential exacerbation of respiratory symptoms and increased mortality rates associated with this condition, particularly among older adults. Although rare, coinfections have high lethality, especially among older adults (Hanada et al., 2018; Amin-Chowdhury et al., 2021; Zhu et al., 2022). 

Invasive pneumococcal disease has been identified as the first documented coinfection in a patient with COVID-19, highlighting the importance of further investigations into this potential association (Ayad et al., 2021).

The variants of SARS-CoV-2 pose additional challenges for pandemic containment, notably due to their potential to increase transmission rates, elevate the risk of reinfection, and reduce the efficacy of interventions such as monoclonal antibodies and vaccination, attributed to mutations in the spike protein. Deepening investigation is crucial for a better understanding of these variants (Tao et al., 2021). The continuous emergence of new variants necessitates adjustments in treatment strategies and vaccine development. Less prevalent variants, albeit more impactful, play a significant role in the severity of COVID-19 cases (Gupta et al., 2023; Khadzhieva et al., 2023).

Among the variants that have emerged since the origin of SARS-CoV-2, several new worrisome lineages have emerged, such as Alpha (B.1.1.7), Beta (B.1.351), Gamma (P.1), Delta (B.1.617.2), and Omicron (B.1.1.529), all of them correlated with an increased capacity for transmission and heightened virulence (Aleem *et al*., 2023).

## Material and Methods

### Collection and processing of biological material for the microbiome

The project was submitted to an Ethics Research Committee through the Plataforma Brasil. It was approved (CAAE: 46363321.2.0000.0018) by the Research Ethics Committee of the Institute of Health Sciences of the Federal University of Pará (CEP-UFPA). All volunteer participants in the research were informed about its content, and objectives, as well as benefits and possible risks. All volunteers who agreed to the terms signed the Informed Consent Form (ICF) and subsequently proceeded to the next stages of the study, including testing for COVID-19 (Panbio COVID-19 Ag Rapid Test Device) and sample collection.

The total number of participants was 45 volunteers, aged between 14 and 50 years. These individuals were divided into two groups: the Positive Group (PG) comprised 14 patients, all with a confirmatory molecular diagnosis for COVID-19. Within this group, seven were female and seven were male, with five individuals aged 21 to 30 years, four aged 31 to 40 years, and five aged over 40 years. These individuals underwent sample collection immediately after the confirmatory test during Sars-CoV-2 infection. 

The Negative Group (NG), consisting of 31 individuals, underwent sample collection immediately after a negative confirmatory test. This group comprised 15 females and 16 males, with four individuals aged 14 to 20 years, 12 aged 21 to 30 years, five aged 31 to 40 years, and 10 aged over 40 years. All individuals in the NG group had not been affected by COVID-19 at the time of collection.

The NG was formed by volunteers and the collections were carried out at the Laboratory of Biological Engineering (ENGBIO), Belém, Pará, Brazil. The samplings for the PG group were performed at the Dr. Humberto Maradei Pereira Hospital, using Rayon swabs through swabbing, one (1) swab for the nasopharynx, in both nostrils and another swab to collect in the oropharyngeal regions, according to the protocol established by the Brazilian Ministry of Health (Ministério da Saúde - Brasil, p. 83). The material was stored in 15 mL conical tubes, without solution (dry storage), and kept in refrigerators at -20°C.

In regards to vaccination, all volunteers in the negative group were vaccinated with at least one dose of the immunizing agent at the time of collection. Conversely, among the group of individuals positive for SARS-CoV-2, only two individuals were not vaccinated (specifically volunteers: PM02281 and PM02281); the remaining were vaccinated with at least one dosage of the immunizing agent.

To isolate the DNA, we first washed the swabs in an Eppendorf tube with 19 µl of PBS buffer and 171 µl of ultra-pure water to remove as many cells as possible. The washing was done manually by carefully rotating the swabs 180º repeatedly. Subsequently, the swabs were discarded, and DNA extraction from the solution was performed following the protocol of the DNeasy Blood & Tissue kit (Qiagen). The total DNA from the nasopharynx and oropharynx was extracted and stored in the same microtube. Quantification of nucleic acids was carried out using the Qubit 2.0 (Invitrogen), and the integrity of the extracted DNA was evaluated by 1% agarose gel electrophoresis. After quality analysis, the DNA was subjected to sequencing.

In addition to samples from the nasopharynx and oropharynx, we collected relevant metadata, such as age, presence of COVID-19 symptoms, history of COVID-19 vaccine doses, use of antibiotics in the last 30 days, type of delivery (vaginal or cesarean), and measures to calculate the Body Mass Index (BMI). All metadata is available in the supplementary material (Table S1).

### Sample preparation and sequencing

The sequencing of the 16S rRNA genes was conducted by first amplifying the hypervariable V4 region using primers with Illumina tail, v4 515F (5’ TCGTCGGCAGCGTCAGATGTGTATAAGAGACAGGTGCCAGCMGCCGCGGTAA 3’) and v4 806R (5’GTCTCGTGGGCTCGGAGATGTGTATAAGAGACAGGGACTACHVGGGTWTCTAAT 3’). The reactions were performed in a final volume of 20 µL, containing 12 ng of DNA, 4 µM of each primer, 0.2 mM of dNTPs, 2.5 mM of MgCl2, and 0.5 U of Phusion Hot Start II DNA polymerase.

Two PCRs were conducted, the first to amplify the V4 region of the 16S gene and the second to index the generated fragments. In the first PCR, 25 cycles were performed, and in the second (for indexing), 8 cycles. The amplification conditions for both PCRs were as follows: an initial denaturation at 98°C for 30 s, followed by cycles of 98°C for 30 s, 55°C for 60 s, and 72°C for 30 s, with a final extension at 72°C for 5 min. The resulting amplicons were purified, pooled, quantified, and sequenced using the Illumina MiSeq, using the v2 nano Kit (Illumina, San Diego, CA, USA) (300 cycles), paired-end reads (2x250bp), according to the manufacturer’s instructions. The raw data was submitted to NCBI with accession number PRJNA1078829. 

### Bioinformatics

The procedures adopted for the analysis of 16S rRNA V4 region sequencing involved several essential steps aimed at obtaining accurate results. Initially, the Forward (R1) and Reverse (R2) read files were renamed according to the final standard. Graphics were constructed using the R software (available at https://www.r-project.org/) to observe the quality of these reads after sequencing, without applying any prior trimming or treatments.

The raw data from the microbiota samples were processed using the Divisive Amplicon Denoising Algorithm (DADA2) version 316, as described by Callahan et al. (2016). During this process, low-quality bases, identified with a quality score (Q<20), were removed based on the following parameters: truncLen=c(245, 240 bp), maxN=0, maxEE=c(3,4), truncQ=2, rm.phix=TRUE, compress=TRUE, multithread=FALSE.

Subsequently, the resulting reads were merged, and chimeric sequences were removed to ensure data integrity. Taxonomic assignment was conducted using the Amplicon Sequence Variants (ASV) method, utilizing the SILVA database version 138.

Alpha and beta diversity analyses were performed in R v.4.3.1 using the packages phyloseq v.1.22.3 and vegan v.2.6-4. Alpha diversity indices, including observed ASVs (species richness), Chao1 (estimated richness), Shannon (diversity), and Simpson (evenness), were calculated for each sample. Group comparisons for alpha diversity metrics were performed using Student’s t-test for each index independently: Chao1 (p = 0.595, t-statistic = 0.536), Shannon (p = 0.651, t-statistic = -0.456), and Simpson (p = 0.311, t-statistic = -1.027). No correction for multiple testing was applied to alpha diversity comparisons, as these were considered exploratory analyses of three conceptually related but distinct aspects of diversity (richness, evenness, and diversity). Statistical significance was set at p < 0.05.

For beta diversity analysis, we computed Bray-Curtis dissimilarity matrix based on relative abundances. Rare taxa with relative abundance <1% were removed prior to analysis to reduce noise. Permutational multivariate analysis of variance (PERMANOVA) with 999 permutations was performed using the adonis2 function from the vegan package to test for differences in microbial community composition between groups (PG vs. NG). PERMANOVA results showed no significant difference between groups (F = 0.805, R² = 0.018, p = 0.708), indicating that SARS-CoV-2 infection status explained only 1.8% of the variation in community structure. Prior to interpreting PERMANOVA results, we verified the homogeneity of multivariate dispersions using the betadisper function (PERMDISP test), which confirmed that differences in group dispersion did not confound our findings (p > 0.05). Additional distance matrices (weighted UniFrac and unweighted UniFrac) were also calculated but showed similar patterns and are not presented here. Other graphics such as the rarefaction curve, relative abundance, and Venn diagram were obtained using default package parameters.

To identify bacterial taxa that differed significantly in relative abundance between SARS-CoV-2 positive and negative groups, we performed differential abundance analysis using LEfSe (Linear discriminant analysis Effect Size) (Segata et al., 2011). LEfSe combines statistical significance testing with biological relevance estimation to identify features that are most likely to explain differences between groups. Prior to LEfSe analysis, microbial count data were normalized to relative abundances (expressed as percentages) to account for differences in sequencing depth across samples. Taxa with mean relative abundance <0.1% across all samples were excluded to reduce noise from rare and poorly represented taxa.

LEfSe analysis was performed using a factorial Kruskal-Wallis rank-sum test (alpha = 0.05) to detect features with significant differential abundance between groups, followed by Linear Discriminant Analysis (LDA) to estimate the effect size of each differentially abundant feature. Multiple testing correction was applied using the Benjamini-Hochberg false discovery rate (FDR) method. Taxa were considered significantly differentially abundant if they met the following criteria: FDR-adjusted p-value < 0.05 and LDA score > 2.0. The LDA score represents the logarithmic discriminative magnitude of each feature, with higher absolute values indicating greater biological relevance. Differential abundance testing was performed at multiple taxonomic levels (phylum, class, order, family, and genus) to provide comprehensive characterization of microbiota shifts associated with SARS-CoV-2 infection.

### Ethical Issues

The project was approved in Ethics Research Committee through the Plataforma Brasil (CAAE: 46363321.2.0000.0018) by the Research Ethics Committee of the Institute of Health Sciences of the Federal University of Pará (CEP-UFPA).

## Results

Of the total 45 samples, 23 were from male volunteers, while 22 were from female volunteers. Regarding the presence of COVID-19 disease, we found that 31 individuals belonged to the negative group, of which 16 were male and 15 were female. In the positive group, composed of 14 individuals, the distribution was observed as 7 men and 7 women.

During the sequencing process, two distinct sequencing runs were conducted. In the first run, a total of 3,466,204 reads were recorded, while in the second run, 2,243,170 reads were obtained. Thus, by summing the reads from both runs, we obtain a total of 5,709,374 reads from the sequenced samples.

We analyzed the sequence quality both before and after the detailed filtering process. Using these reads, a rarefaction curve was generated, as presented in the supplementary material (Figure S1). The total number of generated reads was 923,549, with a maximum of 28,155 and a minimum of 7,618 per sample, resulting in an average of 19,649 reads per sample. From the mentioned reads, we identified a total of 3,237 Amplicon Sequence Variants (ASVs), which were employed in the construction and analysis of the results.

The analyses were compared between two distinct groups: the Positive Group (PG), comprising individuals affected by COVID-19, and the Negative Group (NG), comprising individuals not affected by the disease. Furthermore, a differentiated analysis was conducted considering biological sex, taking into account the presence or absence of SARS-CoV-2 infection. The comparison was made between the following subgroups, as an example: Negative Male Individuals (NMI) versus Positive Male Individuals (PMI), and Negative Female Individuals (NFI) versus Positive Female Individuals (PFI).

**Figure 1 -:**
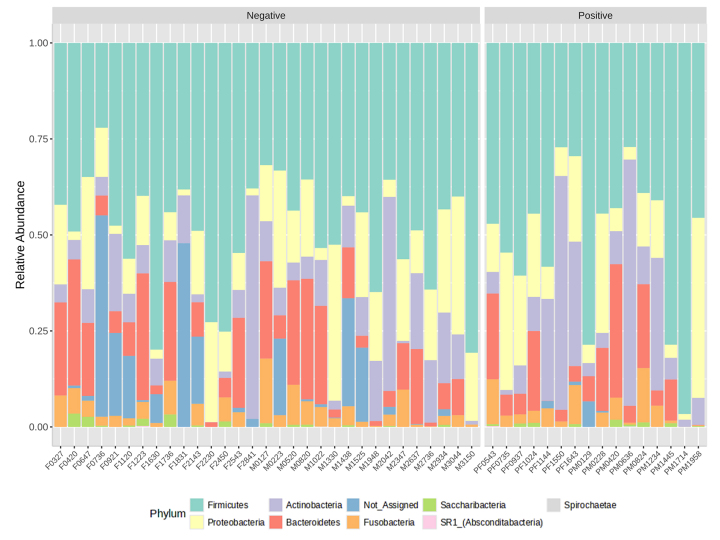
Relative abundance of bacterial phyla in upper respiratory tract samples from SARS-CoV-2 positive and negative individuals. Stacked bar plot showing the relative abundance (%) of bacterial phyla across individual samples from the Negative Group (NG, n=31, left panel) and Positive Group (PG, n=14, right panel). Each vertical bar represents one sample.

The predominant bacterial phyla in both negative and positive groups include Firmicutes, Proteobacteria, Actinobacteria, Bacteroidetes, and Fusobacterium (Figure 1). The most abundant classes in both groups were Bacilli, Actinobacteria, Negativicutes, and Bacteroidia. At the order level, Lactobacillales, Bacillales, and Selenomonadales were prominent, with the family Streptococcaceae being the most abundant, except in positive samples where the family Enterobacteriaceae prevailed. Additionally, the analysis revealed the predominance of the genera *Streptococcus*, *Veillonella*, and *Staphylococcus* in both groups under study. Overall, the assessment of relative abundance showed no significant variations between the negative and positive groups.

Beta diversity analysis using Bray-Curtis dissimilarity revealed high similarity in microbial community composition between groups (Figure S2). PERMANOVA analysis confirmed no significant difference in community structure between PG and NG (F = 0.805, R² = 0.018, p = 0.708), indicating that SARS-CoV-2 infection status explained only 1.8% of the variation in microbial community composition. PERMDISP analysis verified homogeneity of dispersions between groups (p > 0.05), validating the interpretation of PERMANOVA results. The substantial overlap between groups demonstrates that the overall microbiota composition in the upper respiratory tract remains relatively stable despite SARS-CoV-2 infection.

**Figure 2 -:**
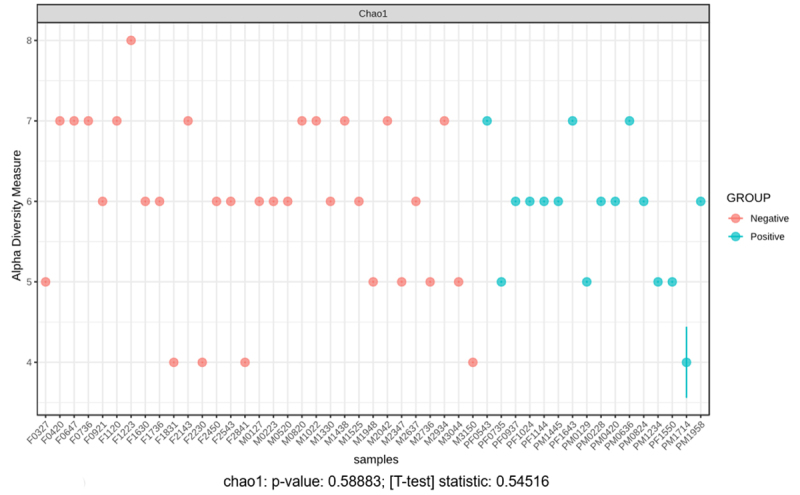
Alpha diversity of upper respiratory tract microbiota in SARS-CoV-2 positive and negative groups. Scatter plot showing Chao1 index values (estimated species richness) for individual samples from the Negative Group (NG, n=31, salmon/coral points) and Positive Group (PG, n=14, turquoise points). Each point represents one sample, with sample IDs labeled on the x-axis. Statistical comparison using Student’s t-test revealed no significant difference between groups (p = 0.595, t-statistic = 0.536).

Alpha diversity analysis revealed no significant differences between groups for any diversity metric (Figure 2). Statistical comparison using Student’s t-test showed that PG and NG did not differ significantly in Chao1 index (estimated richness: p = 0.595, t = 0.536), Shannon index (diversity: p = 0.651, t = -0.456), or Simpson index (evenness: p = 0.311, t = -1.027). While visual inspection showed that some NG samples exhibited greater variability in species richness compared to PG samples, these differences were not statistically significant, suggesting that SARS-CoV-2 infection does not substantially alter overall alpha diversity of the upper respiratory tract microbiota. Detailed alpha diversity values for all samples are available in the supplementary material (Table S2).

**Figure 3 -:**
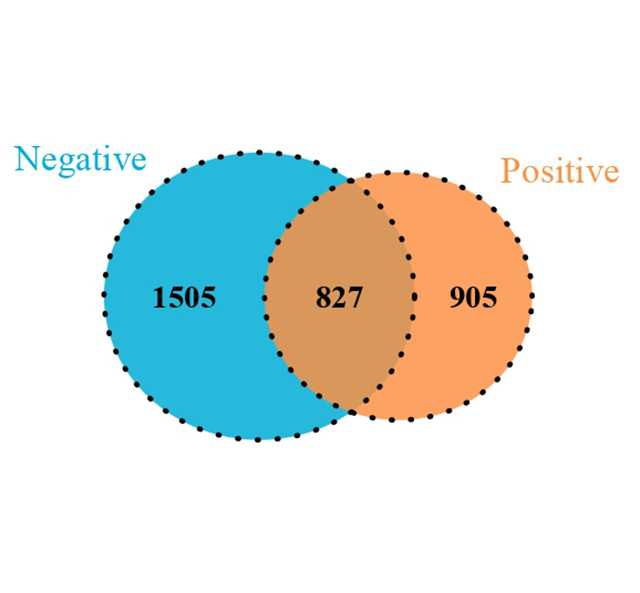
Venn diagram showing shared and unique ASVs between SARS-CoV-2 positive and negative groups. Overlapping circles represent the distribution of Amplicon Sequence Variants (ASVs) between the Negative Group (NG, n=31, turquoise circle, left) and Positive Group (PG, n=14, orange circle, right). The diagram shows that 1,505 ASVs were exclusive to NG, 905 ASVs were exclusive to PG, and 827 ASVs were shared between both groups. The total number of unique ASVs across both groups was 3,237.

A Venn diagram was constructed to conduct a detailed analysis of variations in microbial communities (Figure 3). This facilitated the observation of distinct and shared Amplicon Sequence Variants (ASVs) between the positive and negative groups. Among the obtained results, 1,505 ASVs were exclusively identified in the negative group, 905 belonged to the positive group, and 827 clusters were shared between the negative and positive samples.

LEfSe differential abundance analysis identified several bacterial taxa that were significantly enriched or depleted between positive and negative groups (Table S2). Among the phyla showing significant differences, Actinobacteria, Proteobacteria, and Bacteroidetes were notable. Within Proteobacteria, the Neisseriaceae family was significantly enriched in the positive group (LDA score > 2.0, FDR-adjusted p < 0.05), particularly the genus *Neisseria*, represented by multiple ASVs (ASV_703, ASV_649, ASV_650, ASV_730). These differences may indicate a specific bacterial response associated with SARS-CoV-2 infection. The identification of differentially abundant taxa suggests that distinct bacterial groups within specific phyla and families may play unique roles in the respiratory microbiota during COVID-19 infection.

In addition to the diagram, a table was generated to analyze the representativity of specific taxa with higher incidence, as well as the frequency of the most common taxa in different samples, aiming to facilitate the comparison between groups (Table 1). These tables were created for a detailed visualization of the Phyla, Classes, Orders, and Families present in the microbial communities.

**Table 1 -:** Frequency of ASVs corresponding to the main taxonomic groups observed between COVID-19 positive and negative groups. The taxonomic groups were represented in different segments: Phylum; Class; Order; Green: Family.

Core		Positive		Negative	
Phylum	Frequency	Phylum	Frequency	Phylum	Frequency
Firmicutes	279	Firmicutes	262	Firmicutes	480
Bacteroidetes	163	Bacteroidetes	195	Bacteroidetes	317
Actinobacteria	155	Proteobacteria	194	Proteobacteria	201
Proteobacteria	135	Actinobacteria	114	Actinobacteria	140
Fusobacteria	69	Fusobacteria	73	Fusobacteria	78
Saccharibacteria	10	Spirochaetae	18	Saccharibacteria	36
Others	16	Others	30	Others	57
Core		Positive		Negative	
Class	Frequency	Class	Frequency	Class	Frequency
Bacilli	151	Bacteroidia	155	Clostridia	263
Bacteroidia	151	Bacilli	137	Bacteroidia	248
Actinobacteria	149	Actinobacteria	104	Bacilli	135
Gammaproteobacteria	87	Betaproteobacteria	94	Actinobacteria	119
Negativicutes	78	Clostridia	84	Gammaproteobacteria	93
Fusobacteriia	69	Gammaproteobacteria	79	Fusobacteriia	78
Clostridia	46	Fusobacteriia	73	Negativicutes	69
Betaproteobacteria	37	Flavobacteriia	35	Flavobacteriia	66
Flavobacteriia	12	Negativicutes	30	Betaproteobacteria	52
Epsilonproteobacteria	10	Spirochaetes	18	Alphaproteobacteria	27
Others	37	Others	96	Others	355
Core		Positive		Negative	
Order	Frequency	Order	Frequency	Order	Frequency
Bacteroidales	151	Bacteroidales	155	Clostridiales	263
Lactobacillales	107	Lactobacillales	89	Bacteroidales	248
Corynebacteriales	81	Clostridiales	84	Lactobacillales	94
Selenomonadales	78	Neisseriales	76	Fusobacteriales	78
Fusobacteriales	69	Fusobacteriales	73	Selenomonadales	69
Pasteurellales	65	Corynebacteriales	54	Flavobacteriales	66
Clostridiales	46	Bacillales	42	Corynebacteriales	55
Bacillales	44	Pseudomonadales	40	Pasteurellales	54
Neisseriales	31	Flavobacteriales	35	Bacillales	40
Actinomycetales	29	Selenomonadales	30	Neisseriales	39
Others	126	Others	227	Others	499
Core		Positive		Negative	
Family	Frequency	Family	Frequency	Family	Frequency
Prevotellaceae	136	Prevotellaceae	122	Prevotellaceae	218
Corynebacteriaceae	81	Neisseriaceae	76	Lachnospiraceae	111
Streptococcaceae	79	Streptococcaceae	65	Leptotrichiaceae	73
Veillonellaceae	78	Corynebacteriaceae	54	Family XI	70
Pasteurellaceae	65	Lachnospiraceae	41	Veillonellaceae	69
Fusobacteriaceae	35	Leptotrichiaceae	40	Flavobacteriaceae	66
Leptotrichiaceae	34	Flavobacteriaceae	35	Streptococcaceae	55
Neisseriaceae	31	Fusobacteriaceae	33	Corynebacteriaceae	54
FamilyXI	30	Veillonellaceae	30	Pasteurellaceae	54
Actinomycetaceae	29	Moraxellaceae	26	Neisseriaceae	39

## Discussion

The epithelia of human nasosinusal cavities, as well as pulmonary and intestinal tissue, are colonized by a diverse microbial community, playing a crucial role in modulating epithelial development, immune preparation, and contributing to the context of respiratory diseases (De Rudder et al., 2020 ; Cyprian et al., 2021) Microorganisms colonizing the mucosa of the Upper Respiratory Tract (URT) play a fundamental role in promoting respiratory stability. This microbial community is composed of approximately 46% obligatory anaerobes and produces structural ligands and metabolites that interact with the host, exerting influence on the development and progression of chronic respiratory diseases (Sibley et al., 2011; Budden et al., 2019; Guo et al., 2021). In other ecosystems of the human host, the influence of microbiota composition on pathologies has also been observed, such as the esophageal microbiota in gastrointestinal diseases due to microbial molecules such as lipopolysaccharides (LPS) (Zou et al., 2023; Barchi et al., 2024).

The phyla that exhibited greater representativity in our findings align with the observations made by Guo and colleagues (2021), who analyzed the microbiota in healthy individuals. Firmicutes, in particular, are intrinsically associated with the immunological homeostasis of the respiratory mucosa in the early and adult stages of life. This phylum can be influenced by human behavioral factors, such as smoking, and by biological phenomena, such as SARS-CoV-2 infection (Shabaldin, 2017; Li et al., 2019). We observed a reduction in the frequency of Firmicutes when comparing the negative (NG) and positive (PG) groups, which may be related to dysbiosis and suggest impairment in the immune response to viral infection, potentially contributing to progressive complications associated with the disease. In the findings of Mazziotta and colleagues, the ability of DNA viruses to interfere with host immune modulation was observed, as well as a possible efficient response against the pathogen (Mazziotta *et al*., 2021).

There is controversy regarding the modulation of respiratory microbiota in the presence of SARS-CoV-2, as well as throughout COVID-19. While some studies have reported high microbial diversity, others have found low diversity. There are modulatory mechanisms of respiratory microbial composition during infection that affect the magnitude of the disease. These mechanisms involve alterations in innate and adaptive immune responses, including cytokines, lymphocytes, and inflammatory markers (Zhu et al., 2022).

According to Revilla-Guarinos et al. (2014), Firmicutes have developed a “toolkit” that enables them to colonize diverse environments, including abiotic ones such as soil, and biotic environments like the gastrointestinal tract and the human respiratory mucosa. These microorganisms exhibit notable defensive advantages in situations of competition between microbial communities, particularly those producing antimicrobial peptides (AMPs). Firmicutes have developed defense mechanisms against AMPs involving modifications to the cell wall through D-alanylation, and they play a crucial role in specific ATP-binding cassette transporters for peptidic antibiotics.

Studies suggest that Bacteroidetes in the human respiratory tract possess a conserved secretion system for respiratory control, associated with immune dysregulation, systemic diseases, metabolic syndrome, and neurological disorders (Gibiino et al., 2018; Munoz et al., 2020). This microbial group was less representative of positive volunteers than the control group. This variation may have significant impacts on the contribution of the microbiota to respiratory health, especially in unwell individuals.

Among the most representative phyla, there was less variation when comparing the PG and NG. Previous studies investigating the lower respiratory tract identified a significant increase in microorganisms from the Proteobacteria phylum in patients with bronchial asthma, contrasting with a reduction in Bacteroidetes. Proteobacteria emerge as a potential microbial signature in various human diseases, encompassing metabolic disorders, inflammatory bowel diseases, and pulmonary conditions, where inflammation plays a central role (Rizzatti et al., 2017).

On the other hand, a study also conducted using nasopharyngeal swab samples from SARS-CoV-2-infected patients and employing next-generation sequencing technology, but utilizing different markers, specifically the hypervariable regions V1-V2-V3 of bacterial 16S rRNA, observed similarities to our findings. It identified a significant decrease in the abundance of Proteobacteria and Fusobacteria in COVID-19 patients compared to the control group (Nardelli et al., 2021).

In general, viral pathologies in the respiratory system tend to impair the local microbiota, resulting in a decrease in the representation of important phyla. The Phylum Frequency Table (Table 1) shows changes in the frequencies of major phyla; however, other groups such as Actinobacteria, Fusobacteria, Spirochaetes, Saccharibacteria, Absconditabacteria, Synergistetes, and Cyanobacteria were also observed, but with minimal variations when comparing the microbiota of the volunteers.

The visualization of taxon abundance and beta diversity analysis in distinct collected groups demonstrates remarkable homogeneity. However, upon examining the table, it is possible to observe subtle variations in crucial groups of the microbial community. Small changes in the major representative phyla may play a crucial role in the microscopic ecosystems of the human upper respiratory tract, especially considering that this is the point of entry and lodging for SARS-CoV-2 in chronic infections.

In microbial communities, higher taxonomic ranks such as phyla may provide significant insights into ecological composition; however, subtle variations in diversity are often encapsulated at lower taxonomic levels. By analyzing the lower taxonomic levels presented in Table 1, we observe a sustained higher frequency of taxa in the positive group, with an interesting difference concerning the families present between groups. The Prevotellaceae family is found in both communities, with a higher frequency in the group not exposed to the virus. On the other hand, we note a greater relevance of the Neisseriaceae and Streptococcaceae families in communities with viral contact compared to the healthy group.

Variations in bacterial diversity in the upper respiratory tract may exhibit a potential for reduction as the disease progresses, as evidenced by the particularly significant decrease in Neisseria bacteria in cases of greater severity of the condition (Merenstein et al., 2022). These observations emphasize the importance of considering lower taxonomic levels for a more comprehensive understanding of microbial ecology.

Our positive samples were collected in a hospital setting, excluding the red room (designated for patients with extremely high and unstable conditions requiring immediate interventions for clinical stabilization). This indicates that the patients did not have a severe state of COVID-19 but were hospitalized due to a positive confirmation, being in a moderate or mild state, under observation. Even under these conditions, we observed potential changes in some taxa, such as bacteria “from the Neisseriaceae family. We found through LEfSe analysis that *Neisseria* was significantly enriched in the positive group (LDA score > 2.0, FDR-adjusted p < 0.05), thus promoting an overall increase in the relative abundance of Neisseriaceae ASVs in SARS-CoV-2 infected individuals. In line with this, Rosas-Salazar and colleagues (2023) investigated adult individuals diagnosed with the disease, observing that even in mild infections, there is a possibility of decreased bacterial diversity, thus promoting an increase in the abundance of *Neisseriaceae* ASVs.

While they are part of the healthy nasopharyngeal mucosa microflora, some *Neisseriaceae* species, such as *Neisseria meningitidis*, are characterized as common etiological agents in disseminated respiratory tract infections. These can be classified as potential opportunistic human pathogens in cases of co-infection. Therefore, as mentioned by Yamamoto et al. (2021), the microbiome of the human fecal and respiratory tract undergoes changes in patients with COVID-19, showing an increase in the abundance of opportunistic pathogens. However, the relationship between microbiome alterations and the severity of the disease remains uncertain.

We do not rule out the possibility of opportunistic bacteria; however, based on the observed data, these groups were not evident in our study. Conversely, in a similar study, Gao et al (2021), through analogous analyses using 16S rRNA, besides the decrease in diversity, observed the presence of potential opportunistic bacteria such as Leptotrichia, Granulicatella, and Streptococcus, identifying an increase in these groups among COVID-19 patients.

Braun et al. (2021), conducted a similar investigation to this study. Through amplicon analyses of nasopharyngeal samples from 33 individuals, of whom 21 tested positive, it is suggested that, unlike some other viruses where changes in the microbial community composition were observed, SARS-CoV-2 does not exert a significant impact on the microbial inhabitants of the upper respiratory tract, especially in the nasopharyngeal region. On the contrary, the reverse situation is possible. Disruption of respiratory microbiota and structural disturbance are potentially associated factors with the severity of COVID-19, which may compromise the immune response and promote secondary infections (Hernández-Terán et al., 2021).

The potential microbial contribution to the progression and treatment of the disease was analyzed in a significant portion of the volunteer patients in a study conducted by Wang et al. (2021). In this study, significant changes were observed in the intestinal and airway microbiomes, characterized by an increase in pathogenic bacteria and a decrease in beneficial bacteria. Furthermore, alterations in the Upper Respiratory Tract (URT) can serve as biological markers for the diagnosis and prognosis of the disease, as they are associated with increased mortality from COVID-19, as observed in the influence of respiratory viruses affecting the composition of the pulmonary and intestinal microbial community. These alterations also suggest, through the analysis of variations, the development of therapies (He et al., 2020; Ren et al., 2021).

The obtained results allow for an objective observation and analysis of the behavior of the upper respiratory tract microbiota, highlighting potential variations in its biodiversity in response to SARS-CoV-2 infection. Data extraction from the samples enables the study and comparison of the microbiota between groups of healthy individuals and those affected by COVID-19, providing insights for inferences about possible causes related to viral infection.

Our findings demonstrate subtle alterations in the microbial community during infection, similar to the findings in a study involving individuals with both negative and positive cases of mild and moderate severity over a six-month analysis period, where the bacterial community exhibited stability in its diversity. Although these changes may be modest or specific, they can contribute to understanding the complex interaction between viruses, microbiota, and the host (Armstrong et al., 2023).

In our study, all the data obtained were crucial for formulating such conclusions; nevertheless, it is important to highlight certain situations that might have constrained further inferences. We believe that sample size, the inability to detect cytokine levels, continuous monitoring of patients’ clinical status, and the inability to obtain bacterial DNA load through quantitative PCR could serve as limiting factors in our work.

## Supplementary material

The following online material is available for this article:

Table S1 Representative table of the metadata of the analyzed samples.

Table S2 Frequency of taxa in the demonstrated samples, separated by nucleus, positive patients and negative patients.

Figure S1 Rarefaction curves showing species richness across samples.

Figure S2 Beta diversity analysis of upper respiratory tract microbiota based on Bray-Curtis dissimilarity.

## Data Availability

 The raw data was submitted to NCBI with accession number PRJNA1078829.

## References

[B1] Amin-Chowdhury Z, Aiano F, Mensah A, Sheppard CL, Litt D, Fry NK, Andrews N, Ramsay ME, Ladhani SN (2021). Impact of the coronavirus disease 2019 (COVID-19) pandemic on
invasive pneumococcal disease and risk of pneumococcal coinfection with
severe acute respiratory syndrome coronavirus 2 (SARS-CoV-2): Prospective
national cohort Study, England. Clin Infect Dis.

[B2] Armstrong AJS, Horton DB, Andrews T, Greenberg P, Roy J, Gennaro ML, Carson JL, Panettieri RA, Barrett ES, Blaser MJ (2023). Saliva microbiome in relation to SARS-CoV-2 infection in a
prospective cohort of healthy US adults. eBioMedicine.

[B3] Atzrodt CL, Maknojia I, McCarthy RDP, Oldfield TM, Po J, Ta KTL, Stepp HE, Clements TP (2020). A Guide to Covid‐19: A global pandemic caused by the novel
coronavirus SARS‐CoV‐2. FEBS J.

[B4] Ayad S, Alyacoub R, Gergis K, Grossman D, Salamera J (2021). Invasive pneumococcal disease in a patient with Covid-19: A case
report. Cureus.

[B5] Barchi A, Massimino L, Mandarino FV, Vespa E, Sinagra E, Almolla O, Passaretti S, Fasulo E, Parigi TL, Cagliani S (2024). Microbiota profiling in esophageal diseases: Novel insights into
molecular staining and clinical outcomes. Comput Struct Biotechnol J.

[B6] Bosch AATM, Biesbroek G, Trzcinski K, Sanders EAM, Bogaert D (2013). Viral and bacterial interactions in the upper respiratory
tract. PLoS Pathog.

[B7] Braun T, Halevi S, Hadar R, Efroni G, Glick Saar E, Keller N, Amir A, Amit S, Haberman Y (2021). SARS-CoV-2 does not have a strong effect on the nasopharyngeal
microbial composition. Sci Rep.

[B8] Budden KF, Shukla SD, Rehman SF, Bowerman KL, Keely S, Hugenholtz P, Armstrong-James DPH, Adcock IM, Chotirmall SH, Chung KF, Hansbro PM (2019). Functional effects of the microbiota in chronic respiratory
disease. Lancet Respir Med.

[B9] Callahan BJ, McMurdie PJ, Rosen MJ, Han AW, Johnson AJ, Holmes SP (2016). DADA2: High-resolution sample inference from Illumina amplicon
data. Nat Methods.

[B10] Canedo-Marroquín G, Saavedra F, Andrade CA, Berrios RV, Rodríguez-Guilarte L, Opazo MC, Riedel CA, Kalergis AM (2020). SARS-CoV-2: Immune response elicited by infection and development
of vaccines and treatments. Front Immunol.

[B11] Chiu MC, Li C, Liu X, Song W, Wan Z, Yu Y, Huang J, Xiao D, Chu H, Cai J-P (2022). Human nasal organoids model SARS-CoV-2 upper respiratory
infection and recapitulate the differential infectivity of emerging
variants. mBio.

[B12] Clementi N, Ghosh S, De Santis M, Castelli M, Criscuolo E, Zanoni I, Clementi M, Mancini N (2021). Viral respiratory pathogens and lung injury. Clin Microbiol Rev.

[B13] Copeland E, Leonard K, Carney R, Kong J, Forer M, Naidoo Y, Oliver BGG, Seymour JR, Woodcock S, Burke CM (2018). Chronic rhinosinusitis: Potential role of microbial dysbiosis and
recommendations for sampling sites. Front Cell Infect Microbiol.

[B14] Costello EK, Lauber CL, Hamady M, Fierer N, Gordon JI, Knight R (2009). Bacterial community variation in human body habitats across space
and time. Science.

[B15] Cyprian F, Sohail MU, Abdelhafez I, Salman S, Attique Z, Kamareddine L, Al-Asmakh M (2021). SARS-CoV-2 and immune-microbiome interactions: Lessons from
respiratory viral infections. Int J Infect Dis.

[B16] De Rudder C, Calatayud Arroyo M, Lebeer S, Van De Wiele T (2020). Dual and triple epithelial coculture model systems with
donor-derived microbiota and THP-1 macrophages to mimic host-microbe
interactions in the human sinonasal cavities. mSphere.

[B17] De Steenhuijsen Piters WAA, Sanders EAM, Bogaert D (2015). The role of the local microbial ecosystem in respiratory health
and disease. Philos Trans R Soc B Biol Sci.

[B18] Gao M, Wang H, Luo H, Sun Y, Wang L, Ding S, Ren H, Gang J, Rao B, Liu S (2021). Characterization of the human oropharyngeal microbiomes in
SARS‐CoV‐2 infection and recovery patients. Adv Sci.

[B19] Gibiino G, Lopetuso LR, Scaldaferri F, Rizzatti G, Binda C, Gasbarrini A (2018). Exploring bacteroidetes: Metabolic key points and immunological
tricks of our gut commensals. Dig Liver Dis.

[B20] Guo M-Y, Chen H-K, Ying H-Z, Qiu F-S, Wu J-Q (2021). The role of respiratory flora in the pathogenesis of chronic
respiratory diseases. BioMed Res Int.

[B21] Gupta P, Gupta V, Singh CM, Singhal L (2023). Emergence of Covid-19 variants: An update. Cureus.

[B22] Hanada S, Pirzadeh M, Carver KY, Deng JC (2018). Respiratory viral infection-induced microbiome alterations and
secondary bacterial pneumonia. Front Immunol.

[B23] He Y, Wang J, Li F, Shi Y (2020). Main clinical features of Covid-19 and potential prognostic and
therapeutic value of the microbiota in SARS-CoV-2 infections. Front Microbiol.

[B24] Hernández-Terán A, Mejía-Nepomuceno F, Herrera MT, Barreto O, García E, Castillejos M, Boukadida C, Matias-Florentino M, Rincón-Rubio A, Avila-Rios S (2021). Dysbiosis and structural disruption of the respiratory microbiota
in Covid-19 patients with severe and fatal outcomes. Sci Rep.

[B25] Khadzhieva MB, Gracheva AS, Belopolskaya OB, Kolobkov DS, Kashatnikova DA, Redkin IV, Kuzovlev AN, Grechko AV, Salnikova LE (2023). Covid-19 severity: Does the genetic landscape of rare variants
matter?. Front Genet.

[B26] Kumar D, Pandit R, Sharma S, Raval J, Patel Z, Joshi M, Joshi CG (2022). Nasopharyngeal microbiome of Covid-19 patients revealed a
distinct bacterial profile in deceased and recovered
individuals. Microb Pathog.

[B27] Kumpitsch C, Koskinen K, Schöpf V, Moissl-Eichinger C (2019). The microbiome of the upper respiratory tract in health and
disease. BMC Biol.

[B28] Li K, Chen Z, Huang Y, Zhang R, Luan X, Lei T, Chen L (2019). Dysbiosis of lower respiratory tract microbiome are associated
with inflammation and microbial function variety. Respir Res.

[B29] Lotfi M, Hamblin MR, Rezaei N (2020). COVID-19: Transmission, prevention, and potential therapeutic
opportunities. Clin Chim Acta.

[B30] Man WH, De Steenhuijsen Piters WAA, Bogaert D (2017). The microbiota of the respiratory tract: Gatekeeper to
respiratory health. Nat Rev Microbiol.

[B31] Mazziotta C, Pellielo G, Tognon M, Martini F, Rotondo JC (2021). Significantly low levels of IgG antibodies against oncogenic
merkel cell polyomavirus in sera from females affected by spontaneous
abortion. Front Microbiol.

[B32] Merenstein C, Bushman FD, Collman RG (2022). Alterations in the respiratory tract microbiome in Covid-19:
Current observations and potential significance. Microbiome.

[B33] Munoz R, Teeling H, Amann R, Rosselló-Móra R (2020). Ancestry and adaptive radiation of Bacteroidetes as assessed by
comparative genomics. Syst Appl Microbiol.

[B34] Nardelli C, Gentile I, Setaro M, Di Domenico C, Pinchera B, Buonomo AR, Zappulo E, Scotto R, Scaglione GL, Castaldo G (2021). Nasopharyngeal microbiome signature in COVID-19 positive
patients: Can we definitively get a role to fusobacterium
periodonticum?. Front Cell Infect Microbiol.

[B35] Ren L, Wang Y, Zhong J, Li X, Xiao Y, Li J, Yang J, Fan G, Guo L, Shen Z (2021). Dynamics of the upper respiratory tract microbiota and its
association with mortality in Covid-19. Am J Respir Crit Care Med.

[B36] Revilla-Guarinos A, Gebhard S, Mascher T, Zúñiga M (2014). Antimicrobial peptide resistance in Firmicutes. Environ Microbiol.

[B37] Rizzatti G, Lopetuso LR, Gibiino G, Binda C, Gasbarrini A (2017). Proteobacteria: A common factor in human diseases. BioMed Res Int.

[B38] Rosas-Salazar C, Kimura KS, Shilts MH, Strickland BA, Freeman MH, Wessinger BC, Gupta V, Brown HM, Boone HH, Rajagopala SV (2023). Upper respiratory tract microbiota dynamics following Covid-19 in
adults. Microb Genom.

[B39] Segata N, Izard J, Waldron L, Gevers D, Miropolsky L, Garrett WS, Huttenhower C (2011). Metagenomic biomarker discovery and explanation. Genome Biol.

[B40] Shabaldin AV (2017). Upper respiratory dysbiosis in children. Fundam Clin Med.

[B41] Shilts MH, Rosas-Salazar C, Tovchigrechko A, Larkin EK, Torralba M, Akopov A, Halpin R, Peebles RS, Moore ML, Anderson LJ (2016). Minimally invasive sampling method identifies differences in
taxonomic richness of nasal microbiomes in young infants associated with
mode of delivery. Microb Ecol.

[B42] Sibley CD, Grinwis ME, Field TR, Eshaghurshan CS, Faria MM, Dowd SE, Parkins MD, Rabin HR, Surette MG (2011). Culture enriched molecular profiling of the cystic fibrosis
airway microbiome. PLoS One.

[B43] Siegel SJ, Weiser JN (2015). Mechanisms of bacterial colonization of the respiratory
tract. Annu Rev Microbiol.

[B44] Stearns JC (2015). Culture and molecular-based profiles show shifts in bacterial
communities of the upper respiratory tract that occur with
age. ISME J.

[B45] Tao K, Tzou PL, Nouhin J, Gupta RK, De Oliveira T, Kosakovsky Pond SL, Fera D, Shafer RW (2021). The biological and clinical significance of emerging SARS-CoV-2
variants. Nat Rev Genet.

[B46] Wang H, Wang H, Sun Y, Ren Z, Zhu W, Li A, Cui G (2021). Potential associations between microbiome and
Covid-19. Front Med.

[B47] Watelet JB, Cauwenberge PV (1999). Applied anatomy and physiology of the nose and paranasal
sinuses. Allergy.

[B48] Yamamoto S, Saito M, Tamura A, Prawisuda D, Mizutani T, Yotsuyanagi H (2021). The human microbiome and Covid-19: A systematic
review. PloS One.

[B49] Zhu T, Jin J, Chen M, Chen Y (2022). The impact of infection with Covid-19 on the respiratory
microbiome: A narrative review. Virulence.

[B50] Zou Q, Feng L, Cai X, Qian Y, Xu L (2023). Esophageal microflora in esophageal diseases. Front Cell Infect Microbiol.

