# Supplementary material for: Assessment of the bacterial community of the human upper respiratory tract in patients affected by Covid-19
Source: Genet Mol Biol. 2026 Jun 26;49(Suppl 4):e20250076. doi: 10.1590/1678-4685-GMB-2025-0076 (PMC13322582; doi:10.1590/1678-4685-GMB-2025-0076)
Supplement: Table S1 [file 1415-4757-GMB-49-s4-e20250076-s1.pdf]

Genetics and Molecular Biology
https://doi.org/10.1590/1678-4685-GMB-2025-0076

Supplementary Material to "Assessment of the bacterial community of the human upper respiratory tract in patients affected by Covid-19"

Table S1 - Representative table of the metadata of the analyzed samples. Metadata Summary of Study Participants and Sample Characteristics. This table includes demographic, clinical, and lifestyle information associated with each sequencing sample, such as sex, age group, diagnosis status, symptoms, vaccination history, physical activity, antibiotic usage, inhalant exposure, surgical history, birth type, and BMI classification.

ID	GROUP	Sex	Age	Diagnoses	Symptoms	Vaccine	Activity	Antibiotics	Inhalants	Surgery	Birth	BMI
M0127_R1.fastq.gz	Negative	M	2	0	N	Y	N	N	Y	N	C	Overweight
M0127_R2.fastq.gz	Negative	M	2	0	N	Y	N	N	Y	N	C	Overweight
M0223_R1.fastq.gz	Negative	M	2	0	N	Y	Y	N	N	N	N	Normal
M0223_R2.fastq.gz	Negative	M	2	0	N	Y	Y	N	N	N	N	Normal
F0327_R1.fastq.gz	Negative	F	2	0	N	Y	N	N	N	N	C	Normal
F0327_R2.fastq.gz	Negative	F	2	0	N	Y	N	N	N	N	C	Normal
F0420_R1.fastq.gz	Negative	F	1	0	N	Y	N	N	N	N	N	Normal
F0420_R2.fastq.gz	Negative	F	1	0	N	Y	N	N	N	N	N	Normal
M0520_R1.fastq.gz	Negative	M	1	0	N	Y	N	N	N	N	C	Overweight
M0520_R2.fastq.gz	Negative	M	1	0	N	Y	N	N	N	N	C	Overweight
F0647_R1.fastq.gz	Negative	F	4	0	N	Y	Y	N	Y	Y	C	Overweight
F0647_R2.fastq.gz	Negative	F	4	0	N	Y	Y	N	Y	Y	C	Overweight
F0736_R1.fastq.gz	Negative	F	3	0	N	Y	N	N	N	Y	N	Overweight
F0736_R2.fastq.gz	Negative	F	3	0	N	Y	N	N	N	Y	N	Overweight
M0820_R1.fastq.gz	Negative	M	1	0	N	Y	Y	N	N	N	N	Overweight
M0820_R2.fastq.gz	Negative	M	1	0	N	Y	Y	N	N	N	N	Overweight
F0921_R1.fastq.gz	Negative	F	2	0	N	Y	N	N	N	N	C	Overweight
F0921_R2.fastq.gz	Negative	M	2	0	N	Y	Y	N	N	N	N	Overweight
M1022_R1.fastq.gz	Negative	M	2	0	N	Y	N	N	N	N	C	Normal
M1022_R2.fastq.gz	Negative	M	2	0	N	Y	N	N	N	N	C	Normal
F1120_R1.fastq.gz	Negative	F	1	0	N	Y	Y	N	N	N	N	Normal
F1120_R2.fastq.gz	Negative	F	1	0	N	Y	Y	N	N	N	N	Normal
F1223_R1.fastq.gz	Negative	F	2	0	N	Y	N	N	N	N	C	Normal
F1223_R2.fastq.gz	Negative	F	2	0	N	Y	N	N	N	N	C	Normal
M1330_R1.fastq.gz	Negative	M	2	0	N	Y	N	N	N	Y	C	Obese
M1330_R2.fastq.gz	Negative	M	2	0	N	Y	N	N	N	Y	C	Obese
M1438_R1.fastq.gz	Negative	M	3	0	N	Y	Y	Y	N	Y	N	Overweight
M1438_R2.fastq.gz	Negative	M	3	0	N	Y	Y	Y	N	Y	N	Overweight
M1525_R1.fastq.gz	Negative	M	2	0	N	Y	N	N	N	N	N	Under
M1525_R2.fastq.gz	Negative	M	2	0	N	Y	Y	Y	N	Y	N	Overweight
F1630_R1.fastq.gz	Negative	M	2	0	N	Y	Y	N	N	N	C	Overweight
F1630_R2.fastq.gz	Negative	M	2	0	N	Y	Y	N	N	N	C	Overweight
F1736_R1.fastq.gz	Negative	F	3	0	N	Y	Y	Y	N	Y	C	Obese
F1736_R2.fastq.gz	Negative	F	3	0	N	Y	Y	Y	N	Y	C	Obese
F1831_R1.fastq.gz	Negative	F	3	0	N	Y	N	N	N	Y	C	Obese
F1831_R2.fastq.gz	Negative	F	3	0	N	Y	N	N	N	Y	C	Obese

ID	GROUP	Sex	Age	Diagnoses	Symptoms	Vaccine	Activity	Antibiotics	Inhalants	Surgery	Birth	BMI
M1948_R1.fastq.gz	Negative	M	4	0	N	Y	N	Y	N	N	C	Extremly
M1948_R2.fastq.gz	Negative	M	4	0	N	Y	N	Y	N	N	C	Extremly
M2042_R1.fastq.gz	Negative	M	4	0	N	Y	N	N	N	Y	C	Overweight
M2042_R2.fastq.gz	Negative	M	4	0	N	Y	N	N	N	Y	C	Overweight
F2143_R1.fastq.gz	Negative	F	4	0	N	Y	Y	N	N	Y	C	Normal
F2143_R2.fastq.gz	Negative	F	4	0	N	Y	Y	N	N	Y	C	Normal
F2230_R1.fastq.gz	Negative	F	2	0	N	Y	Y	N	N	Y	C	Normal
F2230_R2.fastq.gz	Negative	F	2	0	N	Y	Y	N	N	Y	C	Normal
M2347_R1.fastq.gz	Negative	M	4	0	N	Y	Y	N	N	Y	N	Overweight
M2347_R2.fastq.gz	Negative	M	4	0	N	Y	Y	N	N	Y	N	Overweight
F2450_R1.fastq.gz	Negative	F	4	0	N	Y	N	N	N	Y	N	F
F2450_R2.fastq.gz	Negative	F	4	0	N	Y	N	N	N	Y	N	F
F2543_R1.fastq.gz	Negative	F	4	0	N	Y	N	Y	N	N	N	Normal
F2543_R2.fastq.gz	Negative	F	4	0	N	Y	N	Y	N	N	N	Normal
M2637_R1.fastq.gz	Negative	M	3	0	N	Y	Y	Y	Y	N	N	Overweight
M2637_R2.fastq.gz	Negative	M	3	0	N	Y	Y	Y	Y	N	N	Overweight
M2736_R1.fastq.gz	Negative	M	3	0	N	Y	N	N	N	N	N	Overweight
M2736_R2.fastq.gz	Negative	M	3	0	N	Y	N	N	N	N	N	Overweight
F2841_R1.fastq.gz	Negative	F	4	0	N	Y	N	Y	N	Y	C	Overweight
F2841_R2.fastq.gz	Negative	F	4	0	N	Y	N	Y	N	Y	C	Overweight
M2934_R1.fastq.gz	Negative	M	3	0	N	F	Y	N	N	N	C	Overweight
M2934_R2.fastq.gz	Negative	M	3	0	N	F	Y	N	N	N	C	Overweight
M3044_R1.fastq.gz	Negative	M	4	0	N	Y	N	N	N	Y	N	Obese
M3044_R2.fastq.gz	Negative	M	4	0	N	Y	N	N	N	Y	N	Obese
M3150_R1.fastq.gz	Negative	M	4	1	N	Y	N	N	N	Y	C	Extremly
M3150_R2.fastq.gz	Negative	M	4	1	N	Y	N	N	N	Y	C	Extremly
PM01291_R1.fastq.gz	Positive	M	2	1	Y	Y	Y	Y	N	N	N	Normal
PM01291_R2.fastq.gz	Positive	M	2	1	Y	Y	Y	Y	N	N	N	Normal
PM02281_R1.fastq.gz	Positive	M	2	1	Y	N	N	Y	Y	N	0	F
PM02281_R2.fastq.gz	Positive	M	2	1	Y	N	N	Y	Y	N	0	F
PM04201_R1.fastq.gz	Positive	M	2	2	Y	Y	Y	Y	N	N	C	Obese
PM04201_R2.fastq.gz	Positive	M	2	2	Y	Y	Y	Y	N	N	C	Obese
PF05431_R1.fastq.gz	Positive	F	4	4	Y	Y	N	N	N	Y	C	Overweight
PF05431_R2.fastq.gz	Positive	F	4	4	Y	Y	N	N	N	Y	C	Overweight
PM06361_R1.fastq.gz	Positive	M	3	1	Y	N	Y	N	Y	Y	0	F
PM06361_R2.fastq.gz	Positive	M	3	1	Y	N	Y	N	Y	Y	0	F
PF07351_R1.fastq.gz	Positive	F	3	2	Y	Y	N	N	N	N	C	Normal
PF07351_R2.fastq.gz	Positive	F	3	2	Y	Y	N	N	N	N	C	Normal
PM08241_R1.fastq.gz	Positive	M	2	1	Y	Y	Y	N	N	N	C	Normal
PM08241_R2.fastq.gz	Positive	M	2	1	Y	Y	Y	N	N	N	C	Normal
PF09391_R1.fastq.gz	Positive	F	3	1	N	Y	N	N	N	N	N	F
PF09391_R2.fastq.gz	Positive	F	3	1	N	Y	N	N	N	N	N	F
PF10241_R1.fastq.gz	Positive	F	2	1	N	Y	Y	N	N	N	N	Overweight
PF10241_R2.fastq.gz	Positive	F	2	1	N	Y	Y	N	N	N	N	Overweight
PF11441_R1.fastq.gz	Positive	F	4	2	Y	Y	Y	N	N	Y	N	Normal
PF11441_R2.fastq.gz	Positive	F	4	2	Y	Y	Y	N	N	Y	N	Normal
PM12341_R1.fastq.gz	Positive	M	3	2	N	Y	N	N	N	N	C	Overweight
PM12341_R2.fastq.gz	Positive	M	3	2	N	Y	N	N	N	N	C	Overweight
PF14431_R1.fastq.gz	Positive	M	4	2	Y	Y	Y	N	N	Y	C	Overweight
PF14431_R2.fastq.gz	Positive	M	4	2	Y	Y	Y	N	N	Y	C	Overweight

ID	GROUP	Sex	Age	Diagnoses	Symptoms	Vaccine	Activity	Antibiotics	Inhalants	Surgery	Birth	BMI
PM15451_R1.fastq.gz	Positive	F	4	2	Y	Y	Y	N	N	Y	N	Overweight
PM15451_R2.fastq.gz	Positive	F	4	2	Y	Y	Y	N	N	Y	N	Overweight
PF16501_R1.fastq.gz	Positive	F	4	2	Y	Y	N	N	N	Y	C	Overweight
PF16501_R2.fastq.gz	Positive	F	4	2	Y	Y	N	N	N	Y	C	Overweight

Subtitle
Sex: (M) Male; (F) Female
Age. (1): Age 14 to 20; (2) age from 21 years to 30 years; (3) age from 31 years to 40 years: (4) Age over 41 years.
Symptoms: (Y) Yes; (N) No.
Vaccine: (S) Had a vaccine dose; (N) Did not take the vaccine dose.
Physical activity: (S) Performs frequent physical activity; (N) Does not perform frequent physical activity.
Antibiotic: Considering the last thirty days. (S) Used antibiotics (N) Did not use antibiotics.
Inhalants: (S) Uses inhalants. (N) Does not use inhalants.
Surgery: (S) Have you ever had a surgical procedure. (N) Never had a surgical procedure performed.
Birth: (C) Caesarean. (N) Normal birth.
