# Supplementary material for: Assessment of the bacterial community of the human upper respiratory tract in patients affected by Covid-19
Source: Genet Mol Biol. 2026 Jun 26;49(Suppl 4):e20250076. doi: 10.1590/1678-4685-GMB-2025-0076 (PMC13322582; doi:10.1590/1678-4685-GMB-2025-0076)
Supplement: Table S2 [file 1415-4757-GMB-49-s4-e20250076-s2.pdf]

Genetics and Molecular Biology
https://doi.org/10.1590/1678-4685-GMB-2025-0076

Supplementary Material to "Assessment of the bacterial community of the human upper respiratory tract in patients affected by Covid-19"

Table S2 -Taxonomic distribution of ASVs across Core, Positive, and Negative groups. Frequency of amplicon sequence variants (ASVs) classified at phylum, class, order, and family levels for Core (consistently present), Positive (enriched), and Negative (depleted) groups. Values represent the number of ASVs identified within each taxonomic category.

Core		Positive		Negative		Core		Positive		Negative	
**Phylum**	**Frequency**	**Phylum**	**Frequency**	**Phylum**	**Frequency**	**Class**	**Frequency**	**Class**	**Frequency**	**Class**	**Frequency**
Firmicutes	279	Firmicutes	262	Firmicutes	480	Bacilli	151	Bacteroidia	155	Clostridia	263
Bacteroidetes	163	Bacteroidetes	195	Bacteroidetes	317	Bacteroidia	151	Bacilli	137	Bacteroidia	248
Actinobacteria	155	Proteobacteria	194	Proteobacteria	201	Actinobacteria	149	Actinobacteria	104	Bacilli	135
Proteobacteria	135	Actinobacteria	114	Actinobacteria	140	Gammaproteobacteria	87	Betaproteobacteria	94	Actinobacteria	119
Fusobacteria	69	Fusobacteria	73	Fusobacteria	78	Negativicutes	78	Clostridia	84	Gammaproteobacteria	93
Saccharibacteria	10	Spirochaetae	18	Saccharibacteria	36	Fusobacteriia	69	Gammaproteobacteria	79	Fusobacteriia	78
Others	16	Saccharibacteria	13	Absconditabacteria	20	Clostridia	46	Fusobacteriia	73	Negativicutes	69
		Absconditabacteria	6	Spirochaetae	18	Betaproteobacteria	37	Flavobacteriia	35	Flavobacteriia	66
		Synergistetes	6	Synergistetes	11	Flavobacteriia	12	Negativicutes	30	Betaproteobacteria	52
		Cyanobacteria	5	Tenericutes	8	Epsilonproteobacteria	10	Spirochaetes	18	Alphaproteobacteria	27
		Others	19	Others	196	Others	37	Others	96	Others	355

Core		Positive		Negative		Core		Positive		Negative	
**Order**	**Frequency**	**Order**	**Frequency**	**Order**	**Frequency**	**Family**	**Frequency**	**Family**	**Frequency**	**Family**	**Frequency**
Bacteroidales	151	Bacteroidales	155	Clostridiales	263	Prevotellaceae	136	Prevotellaceae	122	Prevotellaceae	218
Lactobacillales	107	Lactobacillales	89	Bacteroidales	248	Corynebacteriaceae	81	Neisseriaceae	76	Lachnospiraceae	111
Corynebacteriales	81	Clostridiales	84	Lactobacillales	94	Streptococcaceae	79	Streptococcaceae	65	Leptotrichiaceae	73
Selenomonadales	78	Neisseriales	76	Fusobacteriales	78	Veillonellaceae	78	Corynebacteriaceae	54	FamilyXI	70
Fusobacteriales	69	Fusobacteriales	73	Selenomonadales	69	Pasteurellaceae	65	Lachnospiraceae	41	Veillonellaceae	69
Pasteurellales	65	Corynebacteriales	54	Flavobacteriales	66	Fusobacteriaceae	35	Leptotrichiaceae	40	Flavobacteriaceae	66
Clostridiales	46	Bacillales	42	Corynebacteriales	55	Leptotrichiaceae	34	Flavobacteriaceae	35	Streptococcaceae	55
Bacillales	44	Pseudomonadales	40	Pasteurellales	54	Neisseriaceae	31	Fusobacteriaceae	33	Corynebacteriaceae	54
Neisseriales	31	Flavobacteriales	35	Bacillales	40	FamilyXI	30	Veillonellaceae	30	Pasteurellaceae	54
Actinomycetales	29	Selenomonadales	30	Neisseriales	39	Actinomycetaceae	29	Moraxellaceae	26	Neisseriaceae	39
Others	126	Others	227	Others	499	Others	229	Others	383	Others	696
