# Supplementary material for: Assessment of the bacterial community of the human upper respiratory tract in patients affected by Covid-19
Source: Genet Mol Biol. 2026 Jun 26;49(Suppl 4):e20250076. doi: 10.1590/1678-4685-GMB-2025-0076 (PMC13322582; doi:10.1590/1678-4685-GMB-2025-0076)
Supplement: Figure S1 [file 1415-4757-GMB-49-s4-e20250076-s3.pdf]

Genetics and Molecular Biology
https://doi.org/10.1590/1678-4685-GMB-2025-0076

Supplementary Material to "Assessment of the bacterial community of the human upper respiratory tract in patients affected by Covid-19"

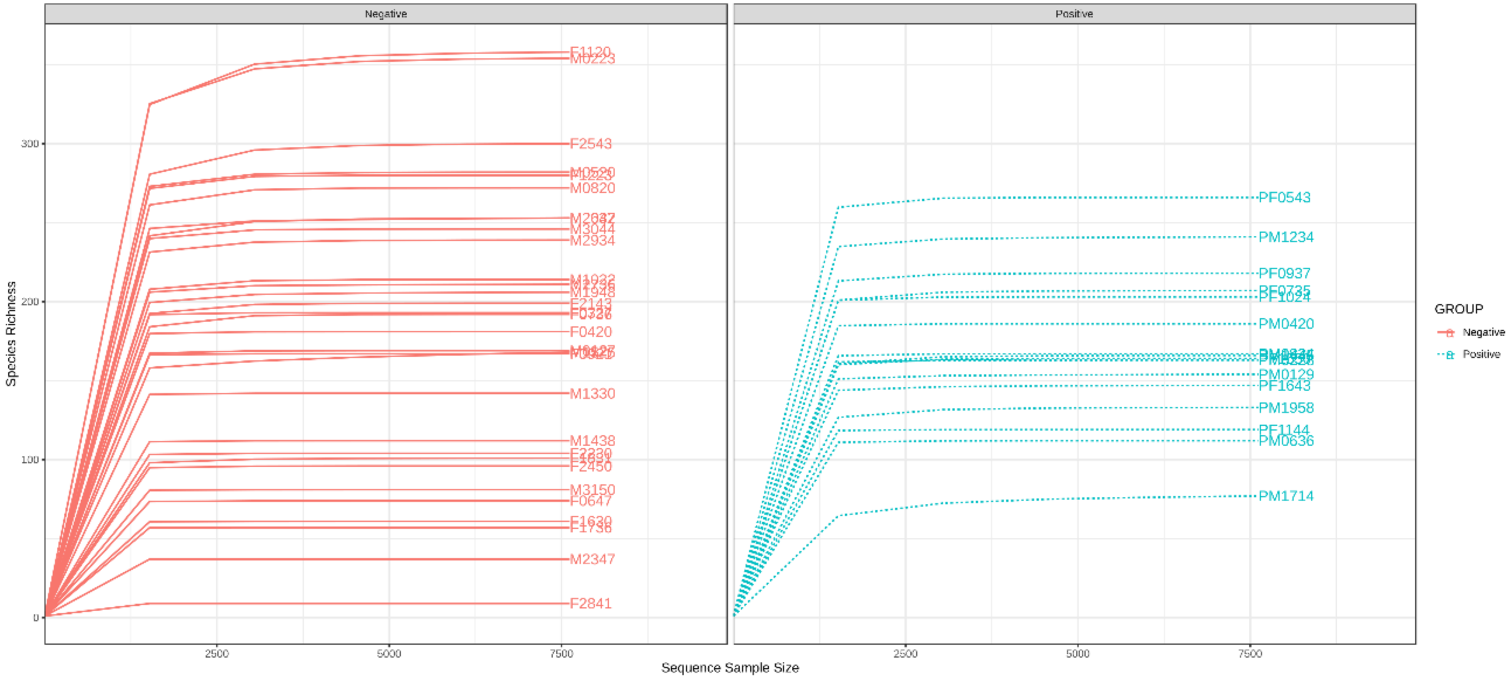

Figure S1 - Rarefaction curves showing species richness across samples. Each curve represents the number of observed species (or ASVs/OTUs) as a function of sequencing depth. The curves tend to plateau, indicating that the sequencing effort was sufficient to capture most of the microbial diversity present in each sample.
