# Supplementary material for: Assessment of the bacterial community of the human upper respiratory tract in patients affected by Covid-19
Source: Genet Mol Biol. 2026 Jun 26;49(Suppl 4):e20250076. doi: 10.1590/1678-4685-GMB-2025-0076 (PMC13322582; doi:10.1590/1678-4685-GMB-2025-0076)
Supplement: Figure S2 [file 1415-4757-GMB-49-s4-e20250076-s4.pdf]

Genetics and Molecular Biology
https://doi.org/10.1590/1678-4685-GMB-2025-0076

Supplementary Material to "Assessment of the bacterial community of the human upper respiratory tract in patients affected by Covid-19"

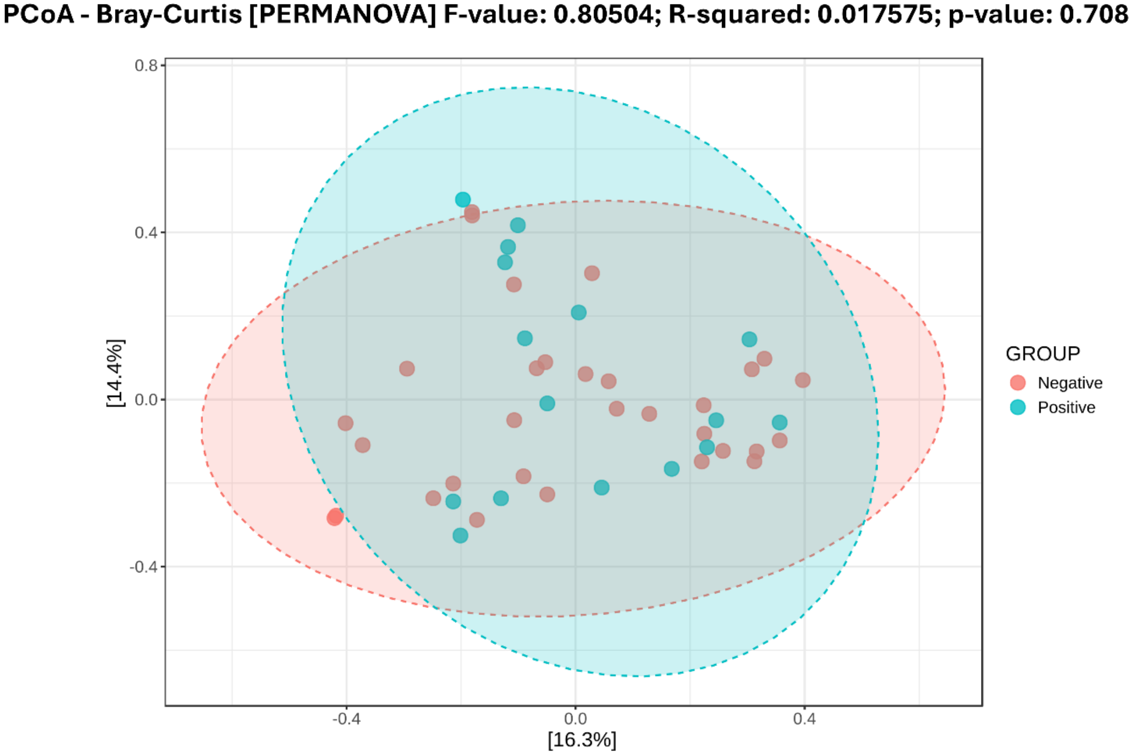

Figure S2 - Beta diversity analysis of upper respiratory tract microbiota based on Bray-Curtis dissimilarity. Principal Coordinates Analysis (PCoA) ordination showing the overall microbial community structure of upper respiratory tract samples from SARS-CoV-2 positive and negative individuals. Each point represents one sample: Positive Group (PG, n=14, turquoise circles) and Negative Group (NG, n=31, salmon/pink circles). The first two principal coordinate axes (PC1 and PC2) explain 16.3% and 14.4% of the total variance, respectively. Ellipses represent 95% confidence intervals around group centroids.
